# Identification and Pharmacological Inactivation of the MYCN Gene Network as a Therapeutic Strategy for Neuroblastic Tumor Cells*[Fn FN2]

**DOI:** 10.1074/jbc.M114.624056

**Published:** 2014-12-04

**Authors:** Olesya Chayka, Cosimo Walter D'Acunto, Odette Middleton, Maryam Arab, Arturo Sala

**Affiliations:** From the ‡ Brunel Institute of Cancer Genetics and Pharmacogenomics, Brunel University London, London UB8 3PH, United Kingdom and; the § Institute of Child Health, University College London, London WC1N 1EH, United Kingdom

**Keywords:** Anticancer Drug, Cancer Biology, Cancer Therapy, Myc (c-Myc), Neuroblastoma, MYCN, shRNA Screen, Synthetic Lethality

## Abstract

The MYC family of transcription factors consists of three well characterized members, c-MYC, L-MYC, and MYCN, deregulated in the majority of human cancers. In neuronal tumors such as neuroblastoma, *MYCN* is frequently activated by gene amplification, and reducing its expression by RNA interference has been shown to promote growth arrest and apoptosis of tumor cells. From a clinical perspective, RNA interference is not yet a viable option, and small molecule inhibitors of transcription factors are difficult to develop. We therefore planned to identify, at the global level, the genes interacting functionally with *MYCN* required to promote fitness of tumor cells facing oncogenic stress. To find genes whose inactivation is synthetically lethal to *MYCN*, we implemented a genome-wide approach in which we carried out a drop-out shRNA screen using a whole genome library that was delivered into isogenic neuroblastoma cell lines expressing or not expressing *MYCN*. After the screen, we selected for in-depth analysis four shRNAs targeting *AHCY*, *BLM*, *PKMYT1*, and *CKS1B.* These genes were chosen because they are directly regulated by MYC proteins, associated with poor prognosis of neuroblastoma patients, and inhibited by small molecule compounds. Mechanistically, we found that BLM and PKMYT1 are required to limit oncogenic stress and promote stabilization of the MYCN protein. Cocktails of small molecule inhibitors of CKS1B, AHCY, BLM, and PKMYT1 profoundly affected the growth of all neuroblastoma cell lines but selectively caused death of *MYCN*-amplified cells. Our findings suggest that drugging the MYCN network is a promising avenue for the treatment of high risk, neuroblastic cancers.

## Teaser

**Background:** Neuroblastic tumors are often addicted to the MYCN protooncogene.

**Results:** Using a genome wide shRNA screen, we have identified key MYCN synthetic lethal genes.

**Conclusion:** Chemical inhibition of the newly identified MYCN synthetic lethal genes selectively kills MYCN-amplified cell lines.

**Significance:** Decoding the MYCN gene network will help to develop drugs for the treatment of neuroblastic tumors with activated MYCN.

## Introduction

There are three well characterized members of the MYC family in mammalian cells, c-MYC, MYCN, and L-MYC (defined hereafter as MYC). Other less studied members are S-MYC and B-MYC. They interact with DNA through a consensus sequence called the E-box (CANNTG) and in concert with the partner MAX facilitate gene transcription. Experimental evidence indicates that MYC and MYCN are functionally interchangeable, with MYCN having a more restricted spatial and temporal role during development (1). The importance of MYC in cell biology is vast. MYC controls key cellular processes including: regulation of cell death (2, 3), angiogenesis (4, 5), metabolism (6), chromatin remodeling (7, 8), and generation of pluripotent stem cells (9). MYC members are proto-oncogenes: on a par with p53 mutation, *MYC* activation is the most frequent molecular alteration observed in human cancer (2). How MYC mechanistically brings about all these functions is still a matter of study, but its classical role as transcriptional activator is being revised in the light of evidence suggesting that MYC is able to modify the chromatin by direct and indirect cross-talk to chromatin modifiers, for example DNA methyl-transferases (7). Along this line, we and others have recently observed that MYCs inhibits the transcription of tumor suppressor genes or microRNAs by physically recruiting the polycomb-repressive complex 2 to promoters in the proximity of MYC-binding sites (10–12). This demonstrates that MYC recruits co-repressor molecules in a sequence-specific manner to induce methylation of histone H3 on lysine 27 and transcriptional repression of specific genes. Given the importance of MYC in the biology of cancer, many clinical researchers are trying to develop drugs that inhibit its activity. Although small molecule inhibitors of MYC are difficult to develop, a dominant-negative variant of MYC, called omomyc, has shown anticancer effects *in vivo*, validating the hypothesis that targeting MYC is highly relevant (13). However, the protein omomyc is unlikely to be used in patients; thus further research is needed to develop clinically viable MYC-targeting drugs. An alternative approach to MYC targeting is to develop drugs that inactivate key MYC partners. For example, it has been shown that MYC-dependent tumors are sensitive to inhibitors of BET family chromatin adaptors and containing bromodomains such as BRD4, required for transcriptional elongation of MYC (14, 15). Furthermore, we and others have shown that inhibiting the MYCN associate EZH2 or other enzymes that modify the chromatin landscape causes reactivation of *CLU* and other potential tumor suppressor genes in cancer cells with therapeutic effects (16–18). This indicates that blocking chromatin modifiers could be of clinical value in MYCN-overexpressing tumors such as neuroblastoma.

Neuroblastoma is the most common extracranial solid tumor in childhood and one of the major causes of cancer death in infancy. Neuroblastoma originates from cells of the neural crest, the embryonal structure that forms the peripheral nervous system. Clinically, we distinguish two types of neuroblastoma: a localized form, usually benign, and a high risk, metastatic form. High risk neuroblastoma has very poor prognosis, and the survival rate after 5 years is only ∼40% despite chemo- and radiotherapy (19). Indeed, metastatic neuroblastoma shows initial response to therapeutic interventions but typically relapses into an incurable form of the disease. It is notable that in the past 20 years there has been no substantial improvement in the outcome of high risk neuroblastoma, suggesting that new avenues of therapy are urgently needed. When activated by amplification in a fraction (∼30%) of neuroblastomas, the protooncogene *MYCN* is a direct cause of the disease. Transgenic expression of *MYCN* in the neuroectoderm causes neuroblastomas in mice with features similar to those seen in the human disease (20). Conversely, inhibition of *MYCN* by antisense approaches results in neuroblastoma regression *in vivo* and *in vitro* (21). Collectively these results demonstrate that *MYCN* is a key driver of tumorigenesis in neuroblastoma, suggesting that therapeutic efforts aimed at inhibiting its expression/activity should have an important clinical relevance.

Activation of *MYC* imposes an oncogenic stress to tumor cells that respond by increasing the expression of genes that enhance cell fitness. The intricate network of genes sustaining the oncogenic activity of *MYC*, the MYC network, if identified, could lead to the development of drugs for cancer therapy. To identify the MYCN network in neuroblastoma we used a global, genome-wide approach in which we carried out an shRNA drop-out screen in isogenic cell lines expressing *MYCN* or not. The prediction was that the introduction of the shRNAs targeting the MYCN network should trigger synthetic lethality in a MYCN-dependent manner. A similar approach has been recently used to identify shRNAs synthetic lethal to c-MYC-overexpressing cells in breast cancer and fibroblasts (22, 23). In another study, the laboratory of Martin Eliers has identified, after the analysis of 97 MYCN target genes, Aurora A as a kinase critically required to stabilize MYCN and whose inhibition by small molecules has a strong impact on MYCN-driven tumors (24, 25).

We describe here the identification of 536 genes whose knockdown is synthetically lethal to MYCN-overexpressing cells. To prioritize candidates, we selected for further analyses genes whose products are inhibited by small molecule drugs, are direct targets of MYCN, and predict poor survival in neuroblastoma patients. Using these criteria, we verified that CKS1B, AHCY, PKMYT1, and BLM could potentially be used as targets for the treatment of *MYCN*-overexpressing tumors.

## MATERIALS AND METHODS

### 

#### 

##### shRNA Screen and Data Analysis

Seven pools of 9,600 shRNAs were prepared from GIPZ Human Whole Genome shRNA Library (Thermo Scientific). The shRNA screen was carried out following published procedures (26) using a multiplicity of infection of 0.3 and a representation of ∼1,000 cellular integrations per shRNA. GIMEN-EMPTY and GIMEN-MYCN cells were infected in triplicate with each pool and harvested at time point 1 (*T* = 1; 48 h after puromycin selection) and at time point 2 (*T* = 2; 2 weeks after time point 1). Genomic DNA from harvested cells was isolated using a blood and cell culture DNA mini kit (Qiagen) following the manufacturer's protocol. Unique barcode sequences were amplified by PCR and purified from agarose gel using Wizard SV gel a PCR clean-up system (Promega, Southampton, UK) following the manufacturer's protocol. Purified PCR products from each cell line and time points were combined and labeled with Cy5 and Cy3 dyes, using the Agilent genomic DNA labeling kit plus, following the Open Biosystem protocol adapted from Agilent oligonucleotide array-based CGH for genomic DNA analysis. Labeled PCR products were then competitively hybridized to custom microarrays containing the barcode sequences. Data extraction was carried out using Aglient's Feature Extraction Software. Analysis was performed using Bioconductor (27) and Limma (28) software. Probes that produced a signal lower than 1.5 times the mean intensity of control probes in at least two of the three replicates were removed. To identify MYCN synthetic lethal shRNAs, the mean log10 Cy5/Cy3 ratios of GIMEN-MYCN replicates was compared with that of GIMEN-EMPTY to derive the log10 ratio difference. Genes with fold change more than 1.5 and *p* values less than 0.05 were considered as potential MYCN synthetic lethal partners.

##### Bioinformatic Analysis

Biofunctions of the candidate shRNAs were assessed with the IPA software (Qiagen). Fisher's Exact test *p* value was used as a scoring method, and the threshold was set at *p* = 0.05. The relationships of MYCN synthetic lethal genes identified in our screen with the three hubs of MYC synthetic lethal genes identified in previous studies (29) was established using the GeneMania software. Indirect interactions were extracted from Pathway commons-Reactome and physical interactions extracted from iRefIndex collection of databases. *In silico* analysis of gene expression in neuroblastoma patients was carried out using the databases Oncomine and Oncogenomics following the instructions and using the tools available at the websites.

##### Lentivirus Production

Lentiviruses were generated by transfecting 5 μg of the pGIPZ-shRNA and packaging plasmids pPAX and pMDG2 into HEK-293FT cells using Lipofectamine 2000 (Invitrogen) following the manufacturer's protocol. Supernatants were harvested 48 h after transfection and filtered through a 0.45-mm filter unit. The sequences and codes of the pGIPZ-shRNA constructs used for shRNA knockdown studies are illustrated in Table 1.

**TABLE 1:** **Details of the shRNA sequences used for the knockdown of MYCN synthetic lethal genes**

Target gene	Sense sequence	Oligonucleotide ID	Barcode ID
CKS 1B	GATGTGCTCTGTATCCAGA	V2LHS_150603	OBS_BC_230698
AHCY	CTCTCCTCCCTAAGAGCTA	V2LHS_112026	OBS_BC_235827
BLM	CTTCCTATGATATTGATAA	V2LHS_89234	OBS_BC_217959
PKMY T1	CGTGTCTAATAAAAAGTAT	V3LHS_644702	
Nonsilencing control	CTCGCTTGGGCGAGAGTAA	RHS4346	

##### Cell Culture

Human embryonic kidney cells 293FT, human Neuroblastoma cell lines SK-N-AS, SH-SY5Y, IMR-32, SK-N-BE(2), LA-N-1, and NB19 were obtained from the American Type Culture Collection (Teddington, Middlesex, UK). GI-M-EN cells were a kind gift from Mirco Ponzoni (Gaslini Hospital, Genova, Italy). SMS-KCNR Neuroblastoma cells were a kind gift from Dr. Andrew Stoker (Institute of Child Health, University College London, London, UK). HEK-293FT, LA-N-1, SK-N-AS, and SH-SY5Y were maintained in DMEM supplemented with 10% heat-inactivated fetal bovine serum, 2 mm glutamine, and sodium pyruvate. IMR-32, NB19, SMS-KCNR, and GI-M-EN were cultured in RPMI medium 1640 containing 10% fetal bovine serum, 2 mm glutamine supplemented with sodium pyruvate and nonessential amino acids. All cell lines were incubated at 37 °C and 5% CO_2_. Stable cell lines expressing the indicated shRNAs were generated by lentiviral transduction in the presence of 8 μg/ml polybrene followed by selection with puromycin (1–4 μg/ml). For drug treatments, cells were plated at a density of 10,000–25,000 cells in 24-well plates in triplicate. After 24 h, the cells were exposed to Fluoxetine (10 mm), ML216 (15 mm), 3-deazaadenosine (20 mm), or PD166285 (0.05 or 0.5 mm) for 24, 48, or 72 h and counted using an hemocytometer or Couness Automated Cell Counter (Invitrogen) after Trypan Blue staining. For growth assays, GIMEN-EMPTY and GIMEN-MYCN cells were seeded into 12-well plates at 10,000 cells/well density in triplicate.

##### Generation of Isogenic Cell Lines Expressing MYCN

CMV-MYCN (16) and the empty pcDNA3.1(+) vector were transfected into GI-M-EN cells using Lipofectamine 2000 (Invitrogen), as described in the manufacturer's protocol. Several G418-resistant clones were analyzed for MYCN expression by Western blot analysis, and the clone expressing the highest levels of MYCN was selected for further experiments.

##### Western Blot Analysis

Neuroblastoma cells were lysed in radioimmune precipitation assay buffer containing 50 mm Tris-HCl (pH 7.4), 150 mm NaCl, 1 mm EDTA, 0.1% sodium deoxycholate, 1% Triton X-100, 0.1% sodium dodecyl sulfate, and a mixture of protease inhibitors (Complete, protease inhibitor mixture tablets; Roche Diagnostics) at 4 °C for 30 min. Cell lysates were mixed with SDS-PAGE loading buffer, loaded onto 8–16% gradient Precise Tris-glycine precast gels (Fisher Scientific) and transferred to PVDF membranes (Fisher Scientific). The antibodies used were MYCN (sc-53993, Santa-Cruz Biotechnology, 1:500 dilution), gamma-H2A.X (phospho S139) (ab11174, Abcam, 1:500 dilution), Actin (sc-1616 Santa Cruz Biotechnology, 1:500 dilution), BLM (A300–110A, Bethyl Laboratories, Cambridge Bioscience, 1:1,000 dilution), PKMYT1 (4282S, Cell Signaling, 1:500 dilution), CKS1B (36-6800, Invitrogen 1:500 dilution), SAHH (H00000191-M07A, Abnova, 1:500 dilution), and c-MYC (phospho T58) (ab28842, Abcam, UK, 1:200 dilution). The membranes were then incubated with appropriate HRP-conjugated secondary antibodies: anti-mouse IgG (NXA931, GE Healthcare, Fisher Scientific), anti-rabbit IgG (NA934, GE Healthcare, Fisher Scientific), or anti-goat IgG (sc-2033, Santa Cruz Biotechnology), all at 1:10,000 dilution. Antibody binding was detected by enhanced chemiluminescence (Fisher Scientific).

##### Annexin V Staining

Apoptosis was detected using an annexin V Alexa Fluor 647 conjugate antibody (640912, BioLegend, Cambridge, UK) following the manufacturer's protocol. Briefly, 2 × 10^6^ cells were harvested and resuspended in 500 μl of annexin V binding buffer (BioLegend) either with or without annexin V antibody (antibody was diluted 1:100 in annexin V binding buffer). After 1 h of incubation on ice, 10 μl of a 200 μg/ml DAPI solution was added to cells before the analysis using a BD LSRII flow cytometer.

##### Propidium Iodide Staining and FACS Analysis

Neuroblastoma cells were fixed on ice with 70% ethanol, then washed twice in phosphate-citrate buffer (0.2 m disodium phosphate and 0.1 m citric acid), and resuspended in PBS containing 2 μg/ml propidium iodide, 0.1% Nonidet P-40, and RNase followed by analysis on BD CyAn ADP flow cytometer.

##### ChIP Assay

ChIP assay was performed as previously described (10, 30). The PCR primers used were: PKMYT1 promoter, 5′-TTATGGACCCAAACACTACGC-3′ and 5′-CGCCAAAAATTCCAAACC-3′; and BLM promoter, 5′-GGCTGAAACAGAAGCATGG-3′ and 5′-TCACCCGTACCCCTCTACAC-3′. Antibodies used in this study were: IgG (sc-2027, Santa Cruz Biotechnology), GAL4 (IgG2A negative control) (Santa Cruz Biotechnology), and MYCN (sc-53993, Santa Cruz Biotechnology).

## RESULTS

### 

#### 

##### Genome-wide shRNA Drop-out Screen

**FIGURE 1.**
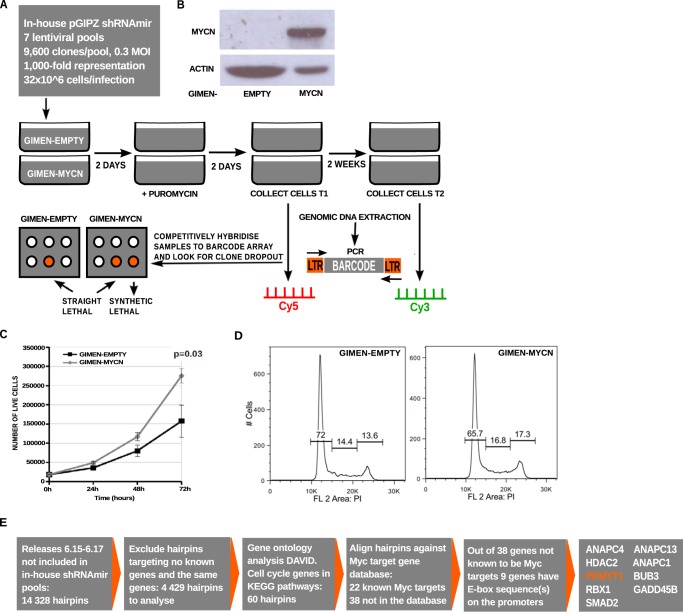
**Genome-wide shRNA screen.** *A*, schematic representation of the shRNA screen strategy. *B*, Western blot analysis demonstrating MYCN overexpression in the cell line stably transfected with the MYCN expression vector. *C*, trypan blue dye exclusion assay illustrating the effect of stable MYCN overexpression on GIMEN-MYCN and GIMEN-EMPTY cells. Cells were counted at the indicated hours from the seeding point. The results represent the means of three experiments performed in triplicate. *Error bars* indicated standard errors. *D*, flow cytometry analysis showing the cell cycle profiles of GIMEN-MYCN and GIMEN-EMPTY cells. *E*, strategy used to identify further synthetic lethal shRNAs based on new releases of the Open Biosystems pGIPZ-shRNAmir lentiviral library.

The shRNA screen was carried out using an shRNA lentiviral library following a protocol described previously (26, 31). The library consists of 67,200 lentiviral shRNA vectors targeting 19,851 human transcripts or 17,700 gene IDs. The library was divided into seven pools, each containing ∼9,600 lentiviral shRNA vectors. Pools were calculated to introduce a single copy of the vector in at least 1,000 cells (Fig. 1*A*). Lentiviral pools were used to infect the control or *MYCN*-expressing GIMEN cell lines (Fig. 1*B*). To generate biological replicates, each pool was delivered in triplicate infections. Genomic DNA was isolated from the infected cells at early and late (2 weeks after puromycin selection) time points. The assumption was that a synthetic lethal shRNA should be depleted by the 2-week time point. We verified that *MYCN* overexpression led to increased growth rates of GIMEN cells as testified by cell growth assays and cell cycle analysis using flow cytometry (Fig. 1, *C* and *D*). These results are in line with previous reports in which *MYCN* overexpression was shown to promote cancer cell proliferation (32, 33).

At the end of the screen, we identified 789 shRNAs depleted in GIMEN-MYCN cells targeting 537 genes (supplemental Data Set S1). To identify additional synthetic lethal shRNA, we inspected 4,429 hairpins that were added in further releases of the Open Biosystem library, focusing on genes implicated in the cell cycle. 60 genes were identified as involved in cell cycle regulation according to the Gene Ontology analysis by DAVID (34, 35), and among these genes, 22 were previously identified as potential MYC-regulated genes. The promoter sequences of the 38 genes not already know as MYC target genes were inspected for the presence of MYCN binding sites using Eukaryotic Promoter Database and TFSEARCH (36, 37). According to this analysis, nine genes contained potential MYCN binding sites in their promoters. Among these, we selected for further analysis PKMYT1 because it is a cell cycle kinase similar to WEE1, whose inactivation was previously demonstrated to be synthetic lethal to c-MYC (23, 38) (Fig. 1*E*).

**FIGURE 2.**
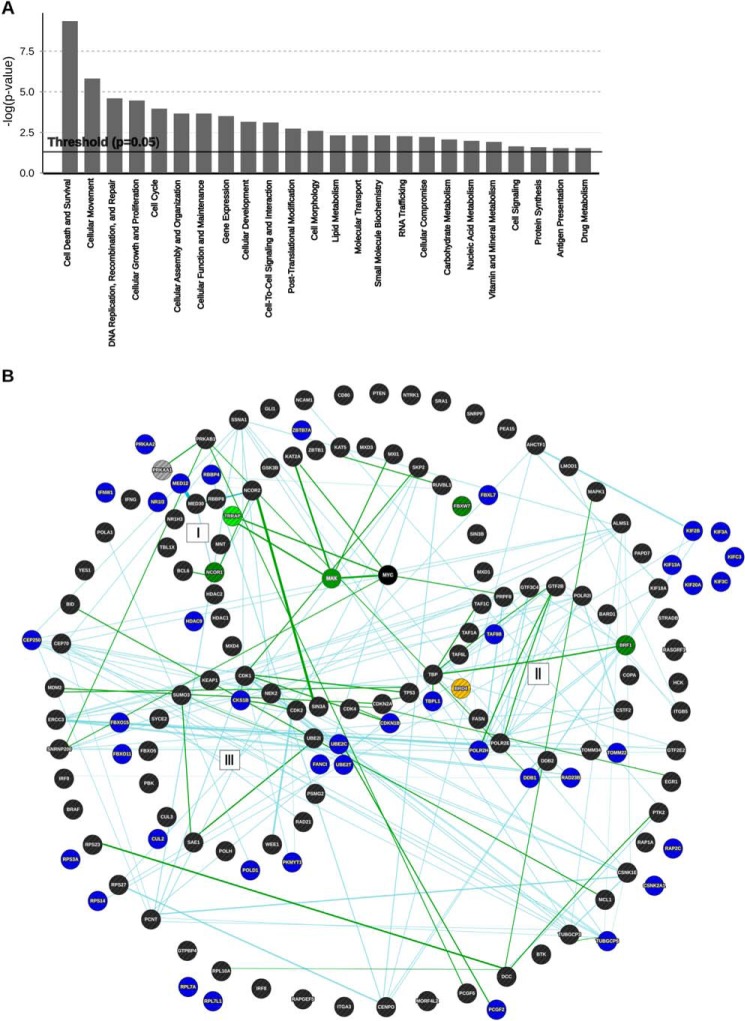
**Bioinformatics analysis of synthetic lethal candidates identified with the shRNA screen.** *A*, molecular and cellular functions of the candidate genes. Fisher's exact test was used as a scoring method, and the threshold was set at *p* = 0.05. *B*, network analysis showing selected MYCN synthetic lethal genes identified in the shRNA screen (*blue*) in relation to the network of MYC synthetic lethal genes identified in previous large scale screens (22, 23). The clustering of the genes was adapted from Fig. 3 in Cermelli *et al.* (29), in which the following functional hubs were identified: (*I*) MYC-MAX network, (*II*) components of transcription initiation and elongation complexes, and (*III*) genes involved in DNA damage repair and cell cycle checkpoints. Genes from the Toyoshima and Kessler screens are depicted in *black*. MYC synthetic lethal genes that intersect between the “core” genes forming a circle around the MYC-MAX network and the Kessler screen are shown in *dark green*; those between the core and the Toyoshima screen are *light green*; those between Kessler and Toyoshima are *yellow*; and those between our screen and Kessler's are *gray*. Direct interactions are shown as *dark green lines*, and indirect interactions are *light blue*.

Pathway analysis showed that the genes identified in the screen are involved in regulation of cell survival, DNA replication and damage, cell cycle, and cellular movements (Fig. 2*A*). According to the Myc Target Gene Database (39), several known MYC target genes score high in the list such as *ABCE1*, *BCL2L12*, *RBBP4*, *NAP1L1*, *EIF2S1*, *POLD1*, *BMP4*, *POLR2H*, *SUCLG1*, *HNRPA1*, *BIRC6*, and *AHCYL1* (supplemental Data Set S1). Notably, a number of the hits identified in our screen are functionally related to the MYC synthetic lethal genes organized in three major hubs recently described by Grandori and co-workers (29) on the basis of published RNAi screens. For example, we detected genes involved in transcriptional initiation and elongation (*DDB1*, *BTAF1*, *TAF9B*, *TBPL1*, and *POLR2H*), transcription and the MYC/MAX network (*HDAC9*, *NR1I3*, *MED23*, *SAP130*, and *MYB*) ubiquitin functions related to the cell cycle checkpoint as well as kinases or other proteins involved in this process (*UBE3C*, *UBE2C*, *UBE20*, *UBE2T*, *RAD21*, *PKMYT1*, *CKS1b*, *CUL2*, *FBXL7*, *FBX040*, *FBX015*, and *FBX011*) (supplemental Data Set S1 and Fig. 2*B*). Notably, we also identified AURKA, a kinase critically required to stabilize the MYCN protein and whose inactivation triggers synthetic lethality in MYCN-amplified neuroblastomas (24).

##### Functional Significance of MYB, PKMYT1, CSK1B, AHCY, and BLM in MYCN-amplified Neuroblastomas

**FIGURE 3.**
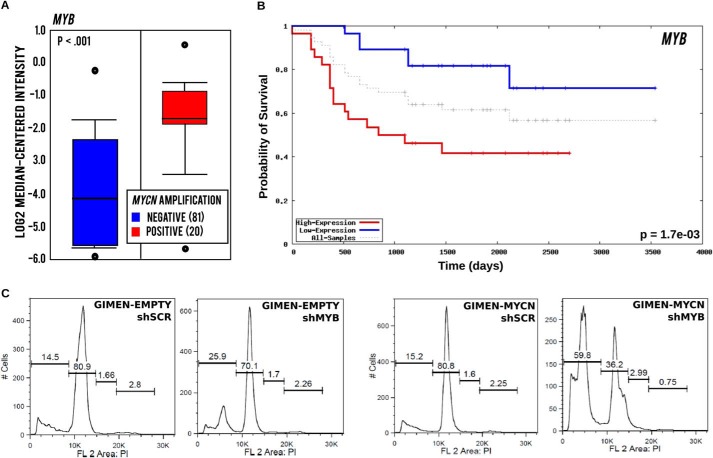
***MYB* expression is increased in *MYCN*-amplified tumors, predicts poor survival of neuroblastoma patients, and is required for survival of *MYCN*-expressing cells.** *A*, box plot showing expression of *MYB* in neuroblastoma tumors with (20 cases) or without (81 cases) *MYCN* amplification. *B*, Kaplan-Meier analysis demonstrating a significant association of *MYB* expression with poor outcome of neuroblastoma patients. *C*, propidium iodide DNA staining and flow cytometry analysis showing increased DNA fragmentation, diagnostic of apoptosis, in GIMEN-MYCN, compared with control, cells after infection with a *MYB* shRNA lentivirus.

The shRNA that was most significantly dropped in *MYCN* positive cells targeted the *MYB* gene, which encodes the c-MYB transcription factor. This is notable, because we have recently shown that the related member *MYBL2*, encoding B-MYB, is a direct target of MYCN and regulates the expression of the MYCN amplicon in a positive regulatory loop. In that study, we demonstrated that ablation of B-MYB triggers synthetic lethality in cells with amplified *MYCN* (40). Similarly to *MYBL2*, *MYB* expression is significantly increased in *MYCN*-amplified neuroblastomas predicting poor survival (Fig. 3, *A* and *B*). To verify whether c-MYB is required for fitness of MYCN-expressing cells, we infected GIMEN and GIMEN-MYCN cells with an shRNA lentiviral vector targeting MYB and observed that this caused massive activation of apoptosis in MYCN, but not non-MYCN, expressing cells (Fig. 3*C*). From a clinical perspective, MYB proteins are not optimal targets because small molecule inhibitors specific for this class of transcription factors have not been yet developed. To prioritize druggable genes and reduce the number of candidates to a manageable number, we used the following criteria: (*a*) the genes are, or have the potential to be, direct MYC targets; (*b*) the genes have prognostic value in neuroblastoma; and (*c*) inhibitors are readily available. We therefore selected *AHCY*, *BLM*, *PKMYT1*, and *CKS1B* for further analysis.

The adenosylhomocysteinase (*AHCY*) gene encodes *S*-adenosylhomocysteine hydrolase (SAHH)^2^ that metabolizes S-adenosyl homocysteine, an inhibitor of methylation reactions. It was shown previously that *AHCY* is a direct c-MYC target gene and that SAHH is critical for c-MYC metabolic effects and tumorigenic activity (41). The SAHH inhibitor 3-deazadenosine (3-DAZA) inhibits methylation reactions and has been shown to possess antiretroviral activity in preclinical experiments (42, 43).

*CKS1B* encodes a cyclin-dependent kinase binding protein that plays a crucial role in cell-cycle regulation. *CKS1B* is overexpressed in many malignancies including breast, prostate, cervical cancers, nasopharyngeal carcinoma, and multiple myeloma, where it promotes cell proliferation (44–49). CKS1B is transcriptionally induced by c-MYC and negatively regulates the cyclin-dependent kinase (Cdk) inhibitor p27^Kip1^ in lymphoma, supporting c-MYC tumorigenesis (50). CKS1B has been shown to overcome the DNA damage response barrier triggered by activated oncoproteins, suggesting that in the context of activated MYC its expression could enhance cancer cell fitness (45). A small molecule inhibitor of CKS1B, fluoexetine (also known as Prozac), is an antidepressant widely used in clinics.

**FIGURE 4.**
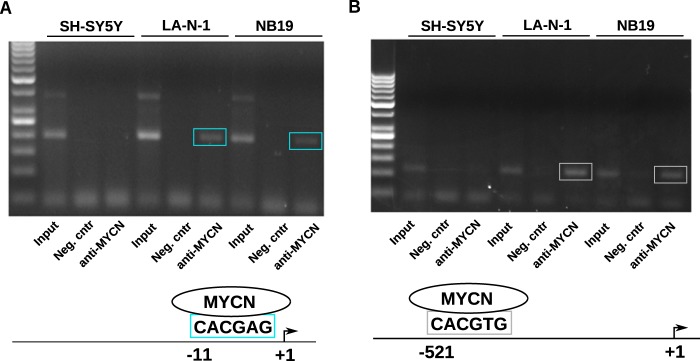
**MYCN binds the *PKMYT1* and *BLM* promoters *in vivo*.** *A*, chromatin immunoprecipitation assays showing binding of MYCN in the region of the *PKMYT1* promoter containing a putative E-box in the proximity of the transcription start site. The binding was detected in the *MYCN*-amplified cell lines LAN1 and NB19 but not in the non-*MYCN*-amplified SHSY5Y cells. *B*, chromatin immunoprecipitation assays showing binding of MYCN in the region of the *BLM* promoter containing a canonical E-box. *Neg. cntr*, negative control.

*PKMYT1* encodes a member of the serine/threonine protein kinase family involved in cell cycle regulation. PKMYT1 inactivates cell division cycle 2 protein (CDK1) by promoting Thr^14^ and Tyr^15^ phosphorylation in concert with the WEE1 kinase (51). This phosphorylation event is critically required for progression of cells into mitosis. Ablation of *WEE1* and *PKMYT1* expression can cause mitotic collapse and apoptosis of cancer cells (38, 52). A small molecule tyrosine kinase inhibitor, PD166285, has been shown to inhibit PKMYT1 at low nanomolar concentrations (53). The *PKMYT1* promoter contains a potential E-box sequence near the transcription start site. ChIP assays confirmed that binding of MYCN could be detected in the *MYCN*-amplified neuroblastoma cell lines NB19 and LA-N-1, whereas it was absent in the non-MYCN-amplified SHSY5Y cell line (Fig. 4*A*).

Bloom syndrome, RecQ helicase protein (BLM), is required to maintain the intra-S-phase checkpoint, and its deregulation may be important for cancer progression (54–57). People carrying mutations in the *BLM* gene are predisposed to different types of cancer and have a shortened lifespan (58). Because accelerated cell division imposed by activated MYC is associated with increased risk of replication errors and DNA damage, it is reasonable to postulate that cancer cells with activated *MYC* are particularly sensitive to reduced RecQ helicases such as BLM and WRN (59–62). Recently, small molecule inhibitors of BLM have been developed that have shown anticancer activity in preclinical studies (63). Examination of the promoter of the *BLM* gene revealed a canonical E-box (CACGTG) located ∼500 bp upstream from the transcriptional start site. We performed ChIP analysis to demonstrate that MYCN binds to the *BLM* promoter in *MYCN*-amplified LA-N-1 and NB19 neuroblastoma cells, whereas the binding was not detected in non-*MYCN*-amplified SH-SY5Y cells (Fig. 4*B*).

**FIGURE 5.**
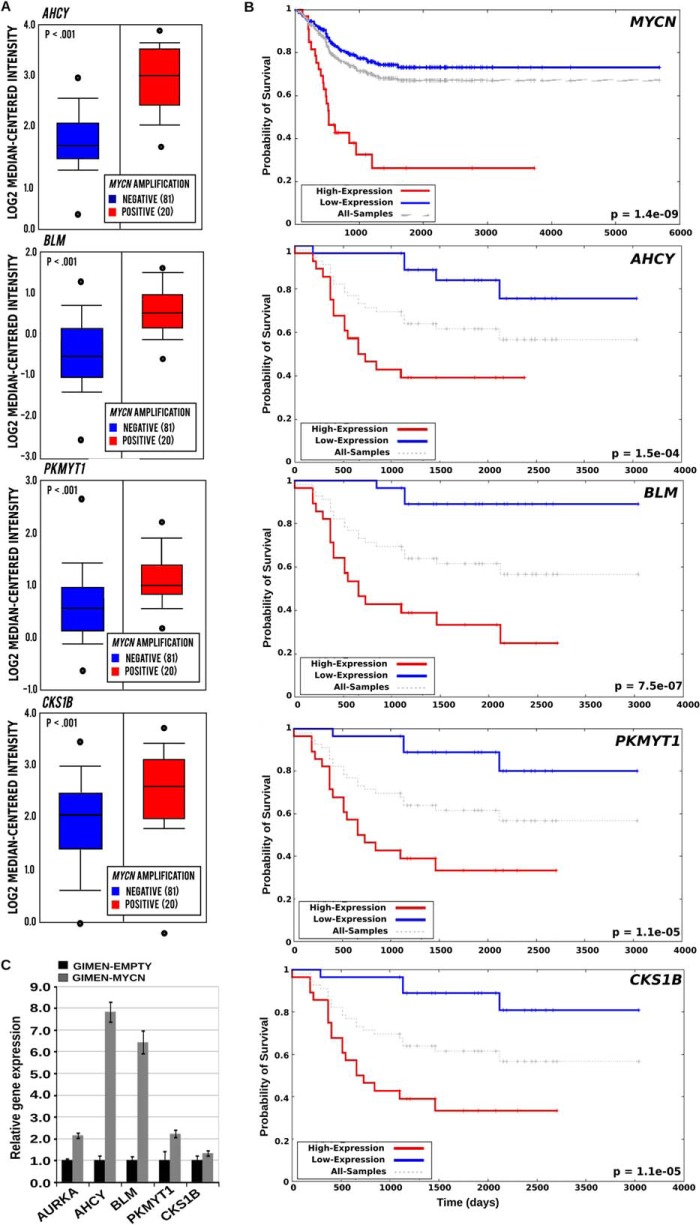
**Expression of AHCY, BLM, PKMYT1, and CKS1B correlates with MYCN expression.** *A*, box plot visualizing the mRNA expression of the different genes in biopsies isolated from neuroblastomas with (20 cases) or without (81 cases) *MYCN* amplification. The data were generated using the tools available in the Oncomine website. *B*, Kaplan-Meier survival curves in patients with high (*red line*) or low (*blue line*) expression of *MYCN*, *AHCY*, *BLM*, *PKMYT1*, or *CKS1B* mRNA expression. The survival curves were generated using the Oncogenomics Neuroblastoma Prognosis database. *C*, Q-PCR analysis demonstrating relative levels of gene expression in the GIMEN-EMPTY and GIMEN-MYCN cell lines. Gene expression was normalized relative to the expression of the housekeeping gene *GAPDH*. The expression levels of the different genes in the control GIMEN-EMPTY cell line were arbitrarily set to 1. Q-PCRs were performed in triplicate. *Error bars* indicate standard deviations.

**FIGURE 6.**
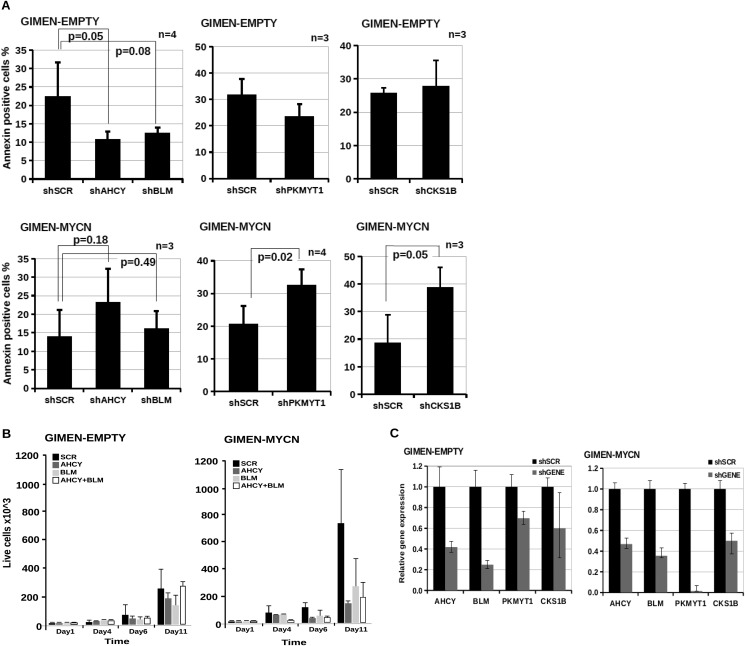
**Down-regulation of *AHCY*, *BLM*, *CKS1B*, and *PKMYT1* increases apoptosis and slows down the growth of neuroblastoma cells overexpressing MYCN.** *A*, apoptosis of GIMEN-EMPTY and GIMEN-MYCN cells infected with lentiviral vectors containing shRNAs targeting *AHCY* (shAHCY), *BLM* (shBLM), *PKMYT1* (shPKMYT1), *CKS1B* (shCKS1B), or scrambled control vector (shSCR) was determined by annexin V staining and flow cytometry. The *error bars* indicate standard deviations, and statistical significance was assessed using Student's *t* test. *B*, trypan blue dye exclusion assay showing effect of shRNA-mediated knockdown of *AHCY* and *BLM* on the growth of GIMEN-EMPTY and GIMEN-MYCN cells. The results represent the means of two independent experiments, each performed in triplicate. *Error bars* indicate standard deviations. *C*, Q-PCR was used to assess gene expression in GIMEN/GIMEN-MYCN cells 1 week after infections with shRNA lentiviral vectors targeting no gene (shSCR) or *AHCY*, *AURKA*, *CKS1B*, *BLM*, or *PKMYT1* (shGENE). Gene expression levels in cells infected with the control virus were set to 1. Q-PCRs were performed in triplicate. *Error bars* indicate standard deviation.

**FIGURE 7.**
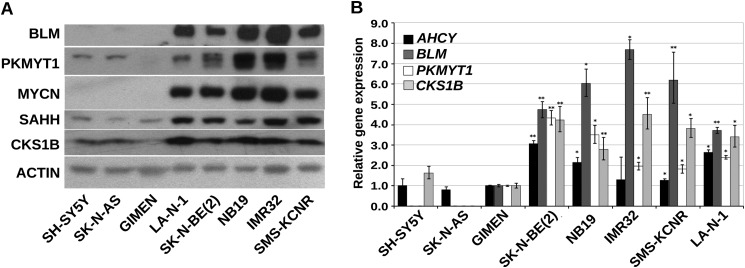
**SAHH, BLM, CKS1B, and PKMYT1 are highly expressed in naturally MYCN-amplified neuroblastoma cell lines.** *A*, Western blot showing SAHH, BLM, PKMYT1, and CKS1B protein expression in a panel of *MYCN*-amplified and nonamplified Neuroblastoma cell lines. Actin was used as a loading control. *B*, Q-PCR demonstrating relative gene expression levels in the MYCN positive or negative Neuroblastoma cell lines. Gene expression levels in the non-MYCN-amplified cell line GIMEN was arbitrarily set to 1, and *GAPDH* expression was used for normalization. The *error bars* indicate standard deviation. *, *p* < 0.005; **, *p* < 0.001 (Student's *t* test *n* = 3).

**FIGURE 8.**
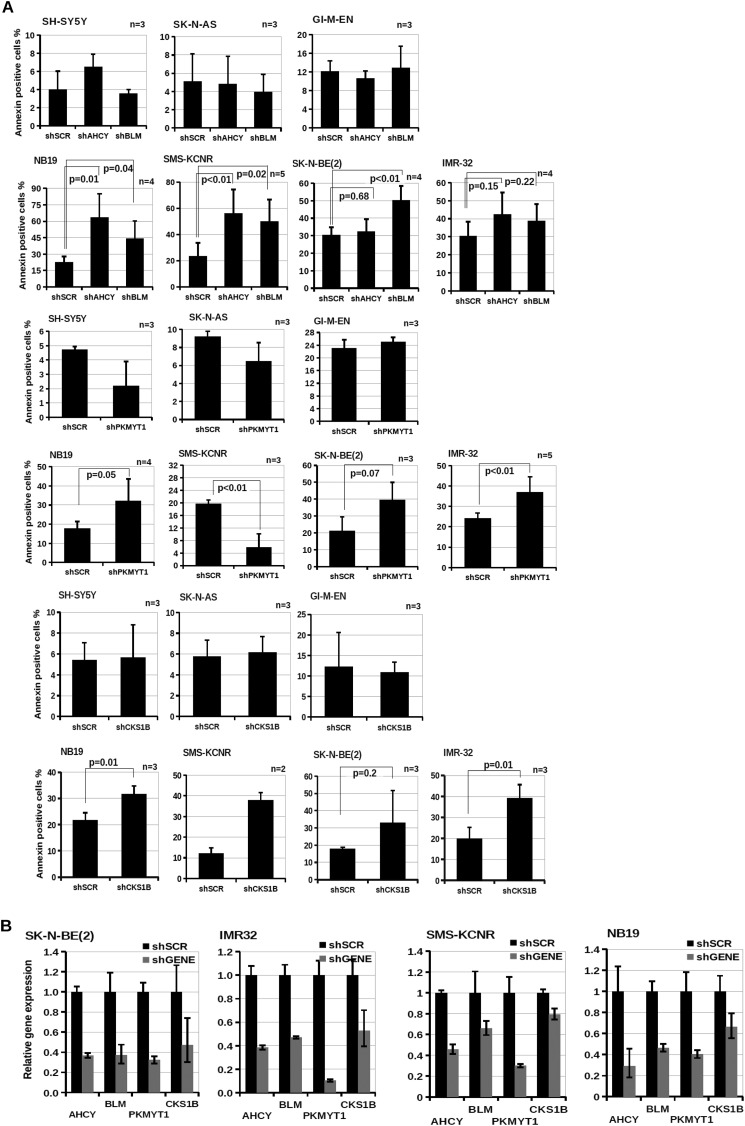
**SAHH, BLM, CKS1B, and PKMYT1 are important for survival of MYCN-amplified neuroblastoma cell lines.** *A*, apoptosis assay was carried out as indicated in Fig. 6*A*. The *error bars* indicate standard deviations, and statistical significance was assessed using Student's *t* test. *B*, Q-PCR was used to assess gene expression in naturally MYCN-amplified neuroblastoma cell lines 1 week after infection with shRNA lentiviral vectors targeting no gene (shSCR) or *AHCY*, *AURKA*, *CKS1B*, *BLM*, or *PKMYT1* (shGENE). Gene expression levels in cells infected with the control virus were set to 1. Q-PCRs were performed in triplicate. *Error bars* indicate standard deviation.

The significance of *BLM*, *AHCY*, *PKMYT1*, and *CKS1B* in the pathogenesis of neuroblastoma is indicated by their elevated expression in *MYCN*-amplified tumor samples and in MYCN-overexpressing cells and their correlation with poor patient survival (Fig. 5, *A–C*). To further validate the selected candidates, we transduced GIMEN-EMPTY and GIMEN-MYCN, with single pGIPZ-shRNAmir lentiviral vectors targeting *PKMYT1*, *AHCY*, *BLM*, and *CKS1B*. After 11 days in culture, GIMEN-MYCN cells infected with the shRNA targeting the candidate genes were more prone to apoptosis or growth arrest than parental cells (Fig. 6, *A* and *B*). Interestingly, concurrent inhibition of AHCY and BLM resulted in additive inhibition of the proliferation of MYCN-expressing cells, suggesting that targeting more than one gene at the time could have clinical value (Fig. 6*B*). As expected, the shRNAs caused reduced gene expression that varied between 90 and 50% in both cell lines (Fig. 6*C*). To confirm that down-regulating the selected genes has the potential to inhibit the survival and/or proliferation of cells with natural amplification of *MYCN*, we first assessed the expression of PKMYT1, BLM, AHCY, and CKS1B at the protein and mRNA levels in a panel of *MYCN*-amplified and nonamplified cell lines. We observed higher expression of the genes in *MYCN*-amplified cells, in line with the hypothesis that they are direct targets of MYCN (Fig. 7, *A* and *B*). Knockdown of the genes caused a selective increase of apoptosis in the majority of MYCN-amplified cells as opposed to cells with no MYCN amplification, confirming that expression of *PKMYT1*, *BLM*, *AHCY*, and *CKS1B* is crucial for the fitness of cells with activated *MYC* (Fig. 8, *A* and *B*).

##### BLM Limits the Oncogenic Stress and PKMYT1 Regulates MYCN Protein Levels in MYCN-amplified Neuroblastoma Cell Lines

**FIGURE 9.**
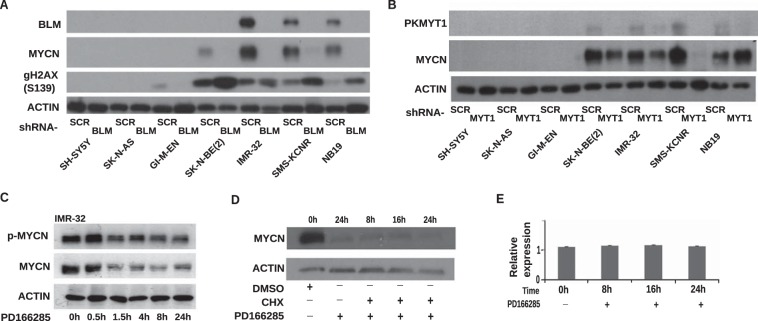
**Knockdown of *BLM* and *PKMYT1* induces genomic stress and destabilize MYCN protein in neuroblastoma cell lines.** *A*, Western blot analysis showing the expression of the indicated proteins after infection of a panel of MYCN-amplified (NB19, SMS-KCNR, IMR32, or SK-N-BE(2)) or nonamplified (GI-M-EN, SK-N-AS, or SH-SY5Y) cells with control or BLM shRNA lentiviruses. *B*, Western blot analysis showing the expression levels of MYCN after shRNA-mediated knockdown of *PKMYT1* in the panel of MYCN-amplified and nonamplified neuroblastoma cells. Expression of Actin was used as a loading control. *C*, Western blot analysis showing the expression of phosphorylated MYCN (p-MYCN) and total MYCN in the neuroblastoma cell lines IMR-32 and NB19 treated with PD166285. Cell lysates were prepared at the indicated time points. Expression of Actin was used as a loading control. *D*, Western blot analysis showing the expression of MYCN in NB19 cells after treatment with PD166285 with or without cycloheximide (*CHX*) or DMSO vehicle. Actin was used as a loading control. *E*, Q-PCR analysis demonstrating unchanged levels of MYCN mRNA after treatment of NB19 cells with PD166285. *Bars* indicate the means of two independent experiments, each performed in triplicate. *Error bars* indicate standard deviations.

It has been reported that inhibition of BLM leads to increased DNA double-stranded breaks and apoptosis of cancer cells (54, 63). To verify the role of BLM in neuroblastoma, we examined the expression of phosphorylated histone γ-H2AX, used as a marker for DNA double-stranded breaks, after infection of neuroblastoma cells with a BLM shRNA. We observed that ablation of *BLM* expression caused increased expression of the γ-H2AX marker in *MYCN* positive cell lines (Fig. 9*A*). Depletion of PKMYT1 caused a drastic reduction of MYCN protein in most of the neuroblastoma cell lines analyzed (Fig. 9*B*). This is consistent with the role of cdk1 as a kinase required to prime MYCN for proteosomal degradation (24). We hypothesized that ablation of PKMYT1 promotes cdk1/GSK3-mediated phosphorylation and degradation of MYCN. Indeed, the PKMYT1 inhibitor PD166285 caused increased phosphorylation of MYCN at threonine 58 followed by sharp degradation of the MYCN protein that did not required *de novo* protein synthesis (Fig. 9, *C* and *D*). As a control, we verified that MYCN mRNA levels were unchanged after the treatments, ruling out an effect on gene expression (Fig. 9*E*).

##### PD166285 and 3-DAZA Cause Selective Apoptosis of MYCN-amplified Cells

**FIGURE 10.**
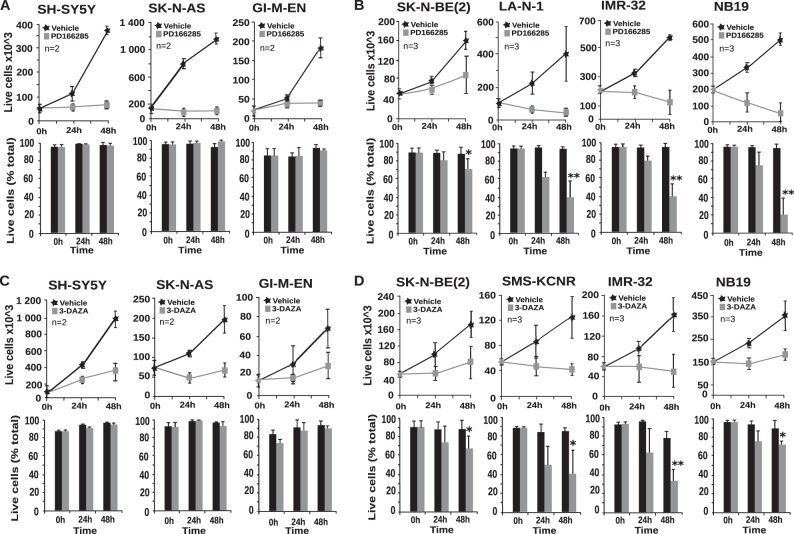
**PD166285 and 3-DAZA trigger synthetic lethality in MYCN-amplified cells.** Nonamplified (*A* and *C*) and MYCN-amplified (*B* and *D*) neuroblastoma cells were exposed to PD166285 and 3-DAZA as indicated. Live/death cells were harvested and counted at the indicated times. The *bars* indicate the mean values of three independent experiments, each performed in triplicate. *Error bars* indicate standard deviations. *, *p* < 0.05; **, *p* < 0.01 (Student's *t* test *n* = 3).

To assess whether chemical inhibitors of PKMYT1 and SAHH have the potential to destabilize the fitness of *MYCN*-amplified cells inducing synthetic lethality, we used PD166285 and 3-DAZA in survival assays. We observed that the compounds inhibited the growth of both *MYCN* positive and negative neuroblastoma cells. However, the drugs selectively killed *MYCN* positive cells (Fig. 10, *A–D*). Analysis by propidium iodide DNA staining and flow cytometry revealed that the drugs caused apoptosis of *MYCN*-positive, but only growth arrest in the G_1_ phase of the cell cycle of *MYCN*-negative cells (data not shown).

##### Concurrent Targeting of PKMYT1, SAHH, BLM, and AHCY as a Possible Therapeutic Approach for MYC-driven Tumors

**FIGURE 11.**
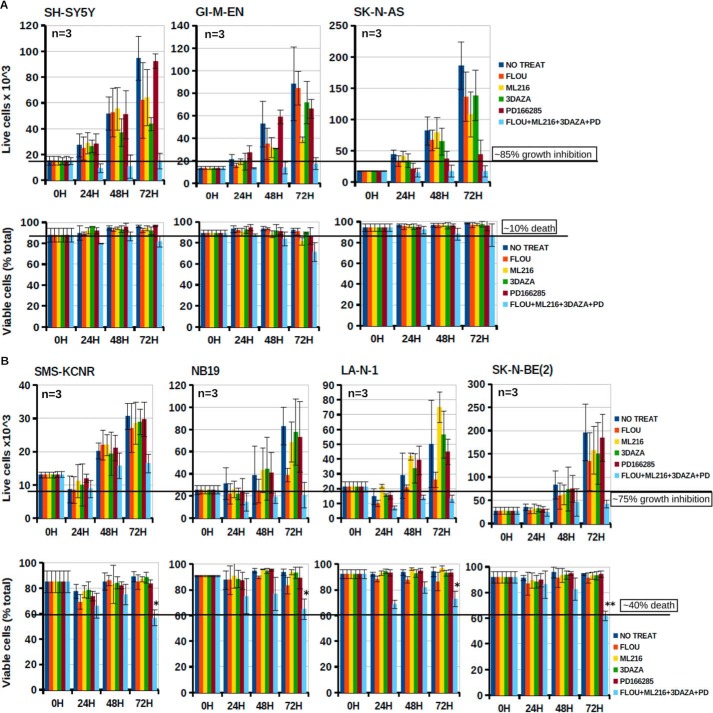
**Combinations of small molecule inhibitors targeting the MYCN interactome suppress neuroblastoma cell proliferation and induce selective killing of MYCN-amplified cells.** Nonamplified (*A*) and MYCN-amplified (*B*) neuroblastoma cell lines were exposed to Flouxetine, ML216, 3-DAZA, and PD166285 alone or in combination as indicated. Live/death cells were harvested at the indicated times. The results represent the means of three independent experiments, each performed in triplicate. *Error bars* indicate standard deviations. *, *p* < 0.01; **, *p* < 0.001 (Student's *t* test).

Combination therapy in which multiple pathways are inhibited at the same time should be advantageous because the cancer cell cannot rely on alternative mechanisms of survival. A further advantage of pharmacological combinations is that the concentration of each agent is lower than that required as a single drug, enhancing specificity and reducing toxicity. We tested the killing effect of PD166285, 3-DAZA, and inhibitors of BLM and CKS1B (ML216 and fluoxetine, respectively). When used as single agents at low micromolar (3-DAZA, ML216, and fluoexetine) or nanomolar (PD166285) concentrations, the drugs were ineffective or only caused a marginal inhibition of cell proliferation. Combining the drugs caused profound inhibition of proliferation of all neuroblastoma cell lines, independently from *MYCN* expression. However, a significant killing effect of the drugs was only observed in *MYCN*-amplified cells (Fig 11, *A* and *B*).

## DISCUSSION

Oncogenic transcription factors are notoriously difficult to target therapeutically, and the hypothesis that we tested in this investigation is whether subverting a MYCN-dependent genetic program could be used as a strategy in treating cancers driven by this protooncogene. To identify key genes and pathways required for survival of cancer cells with activated *MYC*, we carried out an shRNA genome wide drop-out screen in cancer cell lines with or without expression of *MYCN*. Using this method, we identified hundreds of shRNAs depleted in *MYCN*-expressing cells that mainly inactivate genes involved in transcription, cell cycle, and apoptosis. The result of our analysis is consistent with previous RNAi screens in which genes synthetically lethal to c-MYC have been identified in mammalian cells. The Grandori laboratory has recently integrated the results of two major RNAi screen identifying three main hubs. The first hub includes genes involved in transcription initiation and elongation complexes; the second highlights both positive and negative regulators connected to the MYC/MAX network; the third hub includes kinases, ubiquitin, and sumoylation functions related to cell cycle checkpoint and DNA repair (29). Several of the MYCN synthetic lethal genes identified in our screen appear to be related to each of these hubs, in line with the hypothesis that MYCN and c-MYC functions are overlapping, and their role is mainly defined by different spatial and temporal physiopathological contexts. To prioritize interesting candidates, we have manually selected four genes that responded to the following criteria: (*a*) are direct MYC targets, (*b*) have prognostic value, and (*c*) are druggable. The genes selected for further analysis were *AHCY*, *PKMYT1*, *CKS1B*, and *BLM*. PKMYT1 is a serine-threonine kinase that in concert with WEE1 phosphorylates and inactivates cdk1 complexed with cyclinB, regulating entry into mitosis. Interestingly, the MYCN protein is destabilized by phosphorylation of serine 62 by the cdk1/cyclinB kinase required to prime phosphorylation of threonine 58 by gsk3b, initiating proteasomal degradation (24). We showed here that PKMYT1 is required to stabilize the MYCN protein in neuroblastoma cells by reducing T58 phosphorylation by the cdk1/gsk3 kinases, explaining the synthetic lethal effect of PKMYT1 inactivation in *MYCN*-amplified cells. *CKS1B* is overexpressed in several cancers and binds to cdk2, overcoming the replicative barrier imposed by activated oncoproteins. DNA replication stress induced by oncogenes such as Cyclin E or c-MYC can activate an intra-S-phase checkpoint that induces accumulation of inactive cdk2. CKS1B can override the intra-S-phase checkpoint by restoring cdk2 activity (45). Interestingly, inactivation of cdk2 has been previously shown to promote synthetic lethality in cells with amplified *MYCN*, confirming the relevance of this pathway for MYC tumorigenesis (64). BLM is similar to the Werner DNA helicase, WRN, required to limit replication stress imposed by accelerated S-phase imposed by activated oncogenes, including c-MYC (60, 61). Our results support the hypothesis that BLM is critical for the survival of *MYCN*-amplified cells because it limits DNA damage promoted by oncogenic activation, as suggested by the induction of phosphorylated histone γ-H2AX in *MYCN*-amplified cells after depleting BLM expression.

The majority of metastatic, high risk neuroblastomas are characterized by the presence of MYCN amplification. These highly proliferative tumors are treated with chemotherapeutic drugs that are potentially damaging and highly toxic in children. Dissecting the pathways required by cancer cells to tolerate oncogenic stress imposed by activated MYC potentially allows identification of druggable targets that would lead to a more specific and less toxic therapeutic treatments for MYC-driven tumors. To establish, as a proof of principle, that drugging the MYCN network has clinical value, we have used readily available small molecule inhibitors of BLM, CKS1B, PKMYT1, and SAHH. The compounds, either alone or in combination, caused death in the majority of MYCN-amplified cells, suggesting that clinically viable derivatives of these compounds could be used to treat high risk neuroblastomas and other tumors driven by MYC. Of course, the effects of the small molecule inhibitors will need to be validated in appropriate neuroblastoma mouse models prior to any potential clinical application. Mathematical modeling demonstrates that the simultaneous treatment of cancer cells with drugs targeting multiple pathways can improve patients outcome, avoiding drug resistance (65, 66). Our observation that combining pharmacological concentrations of drugs targeting the MYCN network can activate synthetic lethality in *MYCN*-amplified tumor cells suggests that this approach has the potential to be successfully translated in the clinic.

## Supplementary Material

Supplemental Data

## Footnotes

2 The abbreviations used are:

SAHH: *S*-adenosylhomocysteine hydrolase
3-DAZA: 3-deazadenosine.

## References

[1] He S. Liu Z. Oh D. Y. Thiele C. J. (2013) MYCN and the epigenome. Front. Oncol. 3, 1 23373009 10.3389/fonc.2013.00001 PMC3555505

[2] Eilers M. Eisenman R. N. (2008) Myc's broad reach. Genes Dev. 22, 2755–2766 18923074 10.1101/gad.1712408 PMC2751281

[3] Kang J. H. Rychahou P. G. Ishola T. A. Qiao J. Evers B. M. Chung D. H. (2006) MYCN silencing induces differentiation and apoptosis in human neuroblastoma cells. Biochem. Biophys. Res. Commun. 351, 192–197 17055458 10.1016/j.bbrc.2006.10.020 PMC2708968

[4] Chanthery Y. H. Gustafson W. C. Itsara M. Persson A. Hackett C. S. Grimmer M. Charron E. Yakovenko S. Kim G. Matthay K. K. Weiss W. A. (2012) Paracrine signaling through MYCN enhances tumor-vascular interactions in neuroblastoma. Sci. Transl. Med. 4, 115ra3 10.1126/scitranslmed.3002977 PMC3402217 22218692

[5] Dews M. Homayouni A. Yu D. Murphy D. Sevignani C. Wentzel E. Furth E. E. Lee W. M. Enders G. H. Mendell J. T. Thomas-Tikhonenko A. (2006) Augmentation of tumor angiogenesis by a Myc-activated microRNA cluster. Nat. Genet. 38, 1060–1065 16878133 10.1038/ng1855 PMC2669546

[6] Dang C. V. (2011) Therapeutic targeting of Myc-reprogrammed cancer cell metabolism. Cold Spring Harbor Symp. Quant. Biol. 76, 369–374 21960526 10.1101/sqb.2011.76.011296

[7] Brenner C. Deplus R. Didelot C. Loriot A. Viré E. De Smet C. Gutierrez A. Danovi D. Bernard D. Boon T. Pelicci P. G. Amati B. Kouzarides T. de Launoit Y. Di Croce L. Fuks F. (2005) Myc represses transcription through recruitment of DNA methyltransferase corepressor. EMBO J. 24, 336–346 15616584 10.1038/sj.emboj.7600509 PMC545804

[8] Lin C. H. Lin C. Tanaka H. Fero M. L. Eisenman R. N. (2009) Gene regulation and epigenetic remodeling in murine embryonic stem cells by c-Myc. PLoS One 4, e7839 19915707 10.1371/journal.pone.0007839 PMC2773118

[9] Takahashi K. Yamanaka S. (2006) Induction of pluripotent stem cells from mouse embryonic and adult fibroblast cultures by defined factors. Cell 126, 663–676 16904174 10.1016/j.cell.2006.07.024

[10] Corvetta D. Chayka O. Gherardi S. D'Acunto C. W. Cantilena S. Valli E. Piotrowska I. Perini G. Sala A. (2013) Physical interaction between MYCN and polycomb repressive complex 2 (PRC2) in neuroblastoma: functional and therapeutic implications. J. Biol. Chem. 288, 8332–8341 23362253 10.1074/jbc.M113.454280 PMC3605651

[11] Wang L. Zhang X. Jia L. T. Hu S. J. Zhao J. Yang J. D. Wen W. H. Wang Z. Wang T. Zhao J. Wang R. A. Meng Y. L. Nie Y. Z. Dou K. F. Chen S. Y. Yao L. B. Fan D. M. Zhang R. Yang A. G. (2014) c-Myc-mediated epigenetic silencing of MicroRNA-101 contributes to dysregulation of multiple pathways in hepatocellular carcinoma. Hepatology 59, 1850–1863 24002871 10.1002/hep.26720

[12] Zhao X. Lwin T. Zhang X. Huang A. Wang J. Marquez V. E. Chen-Kiang S. Dalton W. S. Sotomayor E. Tao J. (2013) Disruption of the MYC-miRNA-EZH2 loop to suppress aggressive B-cell lymphoma survival and clonogenicity. Leukemia 27, 2341–2350 23538750 10.1038/leu.2013.94 PMC4015113

[13] Soucek L. Jucker R. Panacchia L. Ricordy R. Tatò F. Nasi S. (2002) Omomyc, a potential Myc dominant negative, enhances Myc-induced apoptosis. Cancer Res. 62, 3507–3510 12067996

[14] Fowler T. Ghatak P. Price D. H. Conaway R. Conaway J. Chiang C. M. Bradner J. E. Shilatifard A. Roy A. L. (2014) Regulation of MYC expression and differential JQ1 sensitivity in cancer cells. PLoS One 9, e87003 24466310 10.1371/journal.pone.0087003 PMC3900694

[15] Lovén J. Hoke H. A. Lin C. Y. Lau A. Orlando D. A. Vakoc C. R. Bradner J. E. Lee T. I. Young R. A. (2013) Selective inhibition of tumor oncogenes by disruption of super-enhancers. Cell 153, 320–334 23582323 10.1016/j.cell.2013.03.036 PMC3760967

[16] Chayka O. Corvetta D. Dews M. Caccamo A. E. Piotrowska I. Santilli G. Gibson S. Sebire N. J. Himoudi N. Hogarty M. D. Anderson J. Bettuzzi S. Thomas-Tikhonenko A. Sala A. (2009) Clusterin, a haploinsufficient tumor suppressor gene in neuroblastomas. J. Natl. Cancer Inst. 101, 663–677 19401549 10.1093/jnci/djp063 PMC2720718

[17] Liu T. Tee A. E. Porro A. Smith S. A. Dwarte T. Liu P. Y. Iraci N. Sekyere E. Haber M. Norris M. D. Diolaiti D. Della Valle G. Perini G. Marshall G. M. (2007) Activation of tissue transglutaminase transcription by histone deacetylase inhibition as a therapeutic approach for Myc oncogenesis. Proc. Natl. Acad. Sci. U.S.A. 104, 18682–18687 18003922 10.1073/pnas.0705524104 PMC2141837

[18] Wang C. Liu Z. Woo C. W. Li Z. Wang L. Wei J. S. Marquez V. E. Bates S. E. Jin Q. Khan J. Ge K. Thiele C. J. (2012) EZH2 mediates epigenetic silencing of neuroblastoma suppressor genes CASZ1, CLU, RUNX3, and NGFR. Cancer Res. 72, 315–324 22068036 10.1158/0008-5472.CAN-11-0961 PMC3487161

[19] Brodeur G. M. (2003) Neuroblastoma: biological insights into a clinical enigma. Nat. Rev. Cancer 3, 203–216 12612655 10.1038/nrc1014

[20] Weiss W. A. Aldape K. Mohapatra G. Feuerstein B. G. Bishop J. M. (1997) Targeted expression of MYCN causes neuroblastoma in transgenic mice. EMBO J. 16, 2985–2995 9214616 10.1093/emboj/16.11.2985 PMC1169917

[21] Burkhart C. A. Cheng A. J. Madafiglio J. Kavallaris M. Mili M. Marshall G. M. Weiss W. A. Khachigian L. M. Norris M. D. Haber M. (2003) Effects of MYCN antisense oligonucleotide administration on tumorigenesis in a murine model of neuroblastoma. J. Natl. Cancer Inst. 95, 1394–1403 13130115 10.1093/jnci/djg045

[22] Kessler J. D. Kahle K. T. Sun T. Meerbrey K. L. Schlabach M. R. Schmitt E. M. Skinner S. O. Xu Q. Li M. Z. Hartman Z. C. Rao M. Yu P. Dominguez-Vidana R. Liang A. C. Solimini N. L. Bernardi R. J. Yu B. Hsu T. Golding I. Luo J. Osborne C. K. Creighton C. J. Hilsenbeck S. G. Schiff R. Shaw C. A. Elledge S. J. Westbrook T. F. (2012) A SUMOylation-dependent transcriptional subprogram is required for Myc-driven tumorigenesis. Science 335, 348–353 22157079 10.1126/science.1212728 PMC4059214

[23] Toyoshima M. Howie H. L. Imakura M. Walsh R. M. Annis J. E. Chang A. N. Frazier J. Chau B. N. Loboda A. Linsley P. S. Cleary M. A. Park J. R. Grandori C. (2012) Functional genomics identifies therapeutic targets for MYC-driven cancer. Proc. Natl. Acad. Sci. U.S.A. 109, 9545–9550 22623531 10.1073/pnas.1121119109 PMC3386069

[24] Otto T. Horn S. Brockmann M. Eilers U. Schüttrumpf L. Popov N. Kenney A. M. Schulte J. H. Beijersbergen R. Christiansen H. Berwanger B. Eilers M. (2009) Stabilization of N-Myc is a critical function of Aurora A in human neuroblastoma. Cancer Cell 15, 67–78 19111882 10.1016/j.ccr.2008.12.005

[25] Brockmann M. Poon E. Berry T. Carstensen A. Deubzer H. E. Rycak L. Jamin Y. Thway K. Robinson S. P. Roels F. Witt O. Fischer M. Chesler L. Eilers M. (2013) Small molecule inhibitors of aurora-a induce proteasomal degradation of N-myc in childhood neuroblastoma. Cancer Cell 24, 75–89 23792191 10.1016/j.ccr.2013.05.005 PMC4298657

[26] Schlabach M. R. Luo J. Solimini N. L. Hu G. Xu Q. Li M. Z. Zhao Z. Smogorzewska A. Sowa M. E. Ang X. L. Westbrook T. F. Liang A. C. Chang K. Hackett J. A. Harper J. W. Hannon G. J. Elledge S. J. (2008) Cancer proliferation gene discovery through functional genomics. Science 319, 620–624 18239126 10.1126/science.1149200 PMC2981870

[27] Gentleman R. C. Carey V. J. Bates D. M. Bolstad B. Dettling M. Dudoit S. Ellis B. Gautier L. Ge Y. Gentry J. Hornik K. Hothorn T. Huber W. Iacus S. Irizarry R. Leisch F. Li C. Maechler M. Rossini A. J. Sawitzki G. Smith C. Smyth G. Tierney L. Yang J. Y. Zhang J. (2004) Bioconductor: open software development for computational biology and bioinformatics. Genome Biol. 5, R80 15461798 10.1186/gb-2004-5-10-r80 PMC545600

[28] Smyth G. K. (2004) Linear models and empirical bayes methods for assessing differential expression in microarray experiments. Stat. Appl. Genet. Mol. Biol. 3, Article3 10.2202/1544-6115.1027 16646809

[29] Cermelli S. Jang I. S. Bernard B. Grandori C. (2014) Synthetic lethal screens as a means to understand and treat MYC-driven cancers. Cold Spring Harb. Perspect. Med. 4, a014209 24591535 10.1101/cshperspect.a014209 PMC3935389

[30] Iraci N. Diolaiti D. Papa A. Porro A. Valli E. Gherardi S. Herold S. Eilers M. Bernardoni R. Della Valle G. Perini G. (2011) A SP1/MIZ1/MYCN repression complex recruits HDAC1 at the TRKA and p75NTR promoters and affects neuroblastoma malignancy by inhibiting the cell response to NGF. Cancer Res. 71, 404–412 21123453 10.1158/0008-5472.CAN-10-2627

[31] Silva J. M. Marran K. Parker J. S. Silva J. Golding M. Schlabach M. R. Elledge S. J. Hannon G. J. Chang K. (2008) Profiling essential genes in human mammary cells by multiplex RNAi screening. Science 319, 617–620 18239125 10.1126/science.1149185 PMC2981861

[32] Aubry S. Charron J. (2000) N-Myc shares cellular functions with c-Myc. DNA Cell Biol. 19, 353–364 10882234 10.1089/10445490050043326

[33] Huang R. Cheung N. K. Vider J. Cheung I. Y. Gerald W. L. Tickoo S. K. Holland E. C. Blasberg R. G. (2011) MYCN and MYC regulate tumor proliferation and tumorigenesis directly through BMI1 in human neuroblastomas. FASEB J. 25, 4138–4149 21856782 10.1096/fj.11-185033 PMC3236625

[34] Huang da W. Sherman B. T. Lempicki R. A. (2009) Systematic and integrative analysis of large gene lists using DAVID bioinformatics resources. Nat. Protoc. 4, 44–57 19131956 10.1038/nprot.2008.211

[35] Huang da W. Sherman B. T. Lempicki R. A. (2009) Bioinformatics enrichment tools: paths toward the comprehensive functional analysis of large gene lists. Nucleic Acids Res. 37, 1–13 19033363 10.1093/nar/gkn923 PMC2615629

[36] Heinemeyer T. Wingender E. Reuter I. Hermjakob H. Kel A. E. Kel O. V. Ignatieva E. V. Ananko E. A. Podkolodnaya O. A. Kolpakov F. A. Podkolodny N. L. Kolchanov N. A. (1998) Databases on transcriptional regulation: TRANSFAC, TRRD and COMPEL. Nucleic Acids Res. 26, 362–367 9399875 10.1093/nar/26.1.362 PMC147251

[37] Périer R. C. Junier T. Bonnard C. Bucher P. (1999) The Eukaryotic Promoter Database (EPD): recent developments. Nucleic Acids Res. 27, 307–309 9847211 10.1093/nar/27.1.307 PMC148166

[38] Wang Y. Decker S. J. Sebolt-Leopold J. (2004) Knockdown of Chk1, Wee1 and Myt1 by RNA interference abrogates G_2_ checkpoint and induces apoptosis. Cancer Biol. Ther. 3, 305–313 14726685 10.4161/cbt.3.3.697

[39] Zeller K. I. Jegga A. G. Aronow B. J. O'Donnell K. A. Dang C. V. (2003) An integrated database of genes responsive to the Myc oncogenic transcription factor: identification of direct genomic targets. Genome Biol. 4, R69 14519204 10.1186/gb-2003-4-10-r69 PMC328458

[40] Gualdrini F. Corvetta D. Cantilena S. Chayka O. Tanno B. Raschellà G. Sala A. (2010) Addiction of MYCN amplified tumours to B-MYB underscores a reciprocal regulatory loop. Oncotarget 1, 278–288 21304178 10.18632/oncotarget.138 PMC3248110

[41] Fernandez-Sanchez M. E. Gonatopoulos-Pournatzis T. Preston G. Lawlor M. A. Cowling V. H. (2009) *S*-Adenosyl homocysteine hydrolase is required for Myc-induced mRNA cap methylation, protein synthesis, and cell proliferation. Mol. Cell. Biol. 29, 6182–6191 19805518 10.1128/MCB.00973-09 PMC2786700

[42] Flexner C. W. Hildreth J. E. Kuncl R. W. Drachman D. B. (1992) 3-Deaza-adenosine and inhibition of HIV. Lancet 339, 438 1346708 10.1016/0140-6736(92)90133-n

[43] Mayers D. L. Mikovits J. A. Joshi B. Hewlett I. K. Estrada J. S. Wolfe A. D. Garcia G. E. Doctor B. P. Burke D. S. Gordon R. K. (1995) Anti-human immunodeficiency virus 1 (HIV-1) activities of 3-deazaadenosine analogs: increased potency against 3′-azido-3′-deoxythymidine-resistant HIV-1 strains. Proc. Natl. Acad. Sci. U.S.A. 92, 215–219 7816820 10.1073/pnas.92.1.215 PMC42848

[44] Martinsson-Ahlzén H. S. Liberal V. Grünenfelder B. Chaves S. R. Spruck C. H. Reed S. I. (2008) Cyclin-dependent kinase-associated proteins Cks1 and Cks2 are essential during early embryogenesis and for cell cycle progression in somatic cells. Mol. Cell. Biol. 28, 5698–5709 18625720 10.1128/MCB.01833-07 PMC2546922

[45] Liberal V. Martinsson-Ahlzén H. S. Liberal J. Spruck C. H. Widschwendter M. McGowan C. H. Reed S. I. (2012) Cyclin-dependent kinase subunit (Cks) 1 or Cks2 overexpression overrides the DNA damage response barrier triggered by activated oncoproteins. Proc. Natl. Acad. Sci. U.S.A. 109, 2754–2759 21697511 10.1073/pnas.1102434108 PMC3286935

[46] Lan Y. Zhang Y. Wang J. Lin C. Ittmann M. M. Wang F. (2008) Aberrant expression of Cks1 and Cks2 contributes to prostate tumorigenesis by promoting proliferation and inhibiting programmed cell death. Int. J. Cancer 123, 543–551 18498131 10.1002/ijc.23548 PMC3262990

[47] Wong Y. F. Cheung T. H. Tsao G. S. Lo K. W. Yim S. F. Wang V. W. Heung M. M. Chan S. C. Chan L. K. Ho T. W. Wong K. W. Li C. Guo Y. Chung T. K. Smith D. I. (2006) Genome-wide gene expression profiling of cervical cancer in Hong Kong women by oligonucleotide microarray. Int. J. Cancer 118, 2461–2469 16353136 10.1002/ijc.21660

[48] Lee S. W. Lin C. Y. Tian Y. F. Sun D. P. Lin L. C. Chen L. T. Hsing C. H. Huang C. T. Hsu H. P. Huang H. Y. Wu L. C. Li C. F. Shiue Y. L. (2014) Overexpression of CDC28 protein kinase regulatory subunit 1B confers an independent prognostic factor in nasopharyngeal carcinoma. APMIS 122, 206–214 23879533 10.1111/apm.12136

[49] Bahmanyar M. Qi X. Chang H. (2013) Genomic aberrations in anaplastic multiple myeloma: high frequency of 1q21(CKS1B) amplifications. Leuk. Res. 37, 1726–1728 24169086 10.1016/j.leukres.2013.09.025

[50] Keller U. B. Old J. B. Dorsey F. C. Nilsson J. A. Nilsson L. MacLean K. H. Chung L. Yang C. Spruck C. Boyd K. Reed S. I. Cleveland J. L. (2007) Myc targets Cks1 to provoke the suppression of p27Kip1, proliferation and lymphomagenesis. EMBO J. 26, 2562–2574 17464290 10.1038/sj.emboj.7601691 PMC1868903

[51] Booher R. N. Holman P. S. Fattaey A. (1997) Human Myt1 is a cell cycle-regulated kinase that inhibits Cdc2 but not Cdk2 activity. J. Biol. Chem. 272, 22300–22306 9268380 10.1074/jbc.272.35.22300

[52] Potapova T. A. Sivakumar S. Flynn J. N. Li R. Gorbsky G. J. (2011) Mitotic progression becomes irreversible in prometaphase and collapses when Wee1 and Cdc25 are inhibited. Mol. Biol. Cell 22, 1191–1206 21325631 10.1091/mbc.E10-07-0599 PMC3078080

[53] Rohe A. Erdmann F. Bässler C. Wichapong K. Sippl W. Schmidt M. (2012) *In vitro* and *in silico* studies on substrate recognition and acceptance of human PKMYT1, a Cdk1 inhibitory kinase. Bioorg. Med. Chem. Lett. 22, 1219–1223 22189141 10.1016/j.bmcl.2011.11.064

[54] Seki M. Otsuki M. Ishii Y. Tada S. Enomoto T. (2008) RecQ family helicases in genome stability: lessons from gene disruption studies in DT40 cells. Cell Cycle 7, 2472–2478 18719387 10.4161/cc.7.16.6462

[55] Bachrati C. Z. Hickson I. D. (2003) RecQ helicases: suppressors of tumorigenesis and premature aging. Biochem. J. 374, 577–606 12803543 10.1042/BJ20030491 PMC1223634

[56] Mao F. J. Sidorova J. M. Lauper J. M. Emond M. J. Monnat R. J. (2010) The human WRN and BLM RecQ helicases differentially regulate cell proliferation and survival after chemotherapeutic DNA damage. Cancer Res. 70, 6548–6555 20663905 10.1158/0008-5472.CAN-10-0475 PMC2941797

[57] Horowitz D. P. Topaloglu O. Zhang Y. Bunz F. (2008) Deficiency of Bloom syndrome helicase activity is radiomimetic. Cancer Biol. Ther. 7, 1783–1786 18787401 10.4161/cbt.7.11.6779 PMC2891340

[58] Chu W. K. Hickson I. D. (2009) RecQ helicases: multifunctional genome caretakers. Nat. Rev. Cancer 9, 644–654 19657341 10.1038/nrc2682

[59] Campaner S. Amati B. (2012) Two sides of the Myc-induced DNA damage response: from tumor suppression to tumor maintenance. Cell Division 7, 6 22373487 10.1186/1747-1028-7-6 PMC3310713

[60] Moser R. Toyoshima M. Robinson K. Gurley K. E. Howie H. L. Davison J. Morgan M. Kemp C. J. Grandori C. (2012) MYC-driven tumorigenesis is inhibited by WRN syndrome gene deficiency. Mol. Cancer Res. 10, 535–545 22301954 10.1158/1541-7786.MCR-11-0508 PMC3707802

[61] Robinson K. Asawachaicharn N. Galloway D. A. Grandori C. (2009) c-Myc accelerates S-phase and requires WRN to avoid replication stress. PLoS One 4, e5951 19554081 10.1371/journal.pone.0005951 PMC2694031

[62] Sankar N. Kadeppagari R. K. Thimmapaya B. (2009) c-Myc-induced aberrant DNA synthesis and activation of DNA damage response in p300 knockdown cells. J. Biol. Chem. 284, 15193–15205 19332536 10.1074/jbc.M900776200 PMC2685700

[63] Nguyen G. H. Dexheimer T. S. Rosenthal A. S. Chu W. K. Singh D. K. Mosedale G. Bachrati C. Z. Schultz L. Sakurai M. Savitsky P. Abu M. McHugh P. J. Bohr V. A. Harris C. C. Jadhav A. Gileadi O. Maloney D. J. Simeonov A. Hickson I. D. (2013) A small molecule inhibitor of the BLM helicase modulates chromosome stability in human cells. Chem. Biol. 20, 55–62 23352139 10.1016/j.chembiol.2012.10.016 PMC3558928

[64] Molenaar J. J. Ebus M. E. Geerts D. Koster J. Lamers F. Valentijn L. J. Westerhout E. M. Versteeg R. Caron H. N. (2009) Inactivation of CDK2 is synthetically lethal to MYCN over-expressing cancer cells. Proc. Natl. Acad. Sci. U.S.A. 106, 12968–12973 19525400 10.1073/pnas.0901418106 PMC2695754

[65] Bozic I. Reiter J. G. Allen B. Antal T. Chatterjee K. Shah P. Moon Y. S. Yaqubie A. Kelly N. Le D. T. Lipson E. J. Chapman P. B. Diaz L. A. Jr. Vogelstein B. Nowak M. A. (2013) Evolutionary dynamics of cancer in response to targeted combination therapy. eLife 2, e00747 23805382 10.7554/eLife.00747 PMC3691570

[66] Komarova N. L. Boland C. R. (2013) Cancer: calculated treatment. Nature 499, 291–292 23868257 10.1038/499291a PMC3831845

