# Supplementary material for: Identification and Pharmacological Inactivation of the MYCN Gene Network as a Therapeutic Strategy for Neuroblastic Tumor Cells
Source: J Biol Chem. 2014 Dec 4;290(4):2198–212. doi: 10.1074/jbc.M114.624056 (PMC4303671; doi:10.1074/jbc.M114.624056)
Supplement: Supplemental Data [file supp_290_4_2198__index.html]

Identification and Pharmacological Inactivation of the MYCN Gene Network as a Therapeutic Strategy for Neuroblastic Tumor Cells — MYCN Synthetic Lethal Genes in Neuroblastoma — Supplemental Data 

# Identification and Pharmacological Inactivation of the MYCN Gene Network as a Therapeutic Strategy for Neuroblastic Tumor Cells

## Supplemental Data

**Files in this Data Supplement:**

- Supplemental Data (.xls, 357 KB)
